# Indications et résultats de la chirurgie de résection des bulles d’emphysème pulmonaire

**DOI:** 10.11604/pamj.2018.31.48.16160

**Published:** 2018-09-20

**Authors:** Assane Ndiaye, David Douglas Banga Nkomo, Souleymane Diatta, Papa Salmane Ba, Magaye Gaye, Modibo Doumbia, Pape Adama Dieng, Amadou Gabriel Ciss, Mouhamadou Ndiaye

**Affiliations:** 1 Service de Chirurgie Thoracique et Cardio-vasculaire, Centre Hospitalier National Universitaire de Fann, Dakar, Sénégal; 2 Centre des Urgences de Yaoundé, Yaoundé, Cameroun

**Keywords:** Bullectomie, emphysème, fuites aériennes persistantes, Bullectomy, emphysema, persistent air leaks

## Abstract

La chirurgie d'exérèse des bulles ou bullectomie, principal moyen thérapeutique dans la prise en charge des bulles d'emphysème pulmonaire, est généralement réservée aux patients dont les bulles sont compliquées ou, sont à l'origine d'une dyspnée invalidante. Le but de notre étude était de déterminer les indications de la bullectomie et d'évaluer les résultats de cette chirurgie dans notre service. Nous avons mené une étude rétrospective descriptive de 24 patients (23 hommes et 1 femme), dont la moyenne d'âge était de 49 ans, et qui ont bénéficié d'une bullectomie entre 2004 et 2013. Les données recueillies étaient les facteurs favorisant la survenue d'un emphysème bulleux, les circonstances de découverte de la bulle, les données des examens radiologiques, les données de l'évaluation fonctionnelle respiratoire et cardiovasculaire, les données de la technique de la bullectomie, les données de l'évaluation clinique et fonctionnelle post opératoire. Le taux de morbidité était de 37,5%. La principale complication était la fuite aérienne persistante (7 cas). Un patient est décédé au 2^e^ jour post opératoire suite à une insuffisance respiratoire aiguë. La durée moyenne de suivi était de 26 mois. Durant ce suivi, nous avons observé une amélioration de la dyspnée chez tous les patients et nous n'avons noté aucune complication. La bullectomie est une technique chirurgicale efficace, fiable et sûre qui peut permettre aux patients d'avoir une meilleure qualité de vie pendant quelques années.

## Abstract (en)

Surgical resection of bubbles or bullectomy is the gold standard in the treatment of bubbles arising from pulmonary emphysema. It is usually indicated for patients with complicated bubbles or when they are the underlying cause of disabling dyspnea. This study aims to determine the indications for bullectomy and to evaluate surgical outcomes in our Department. We conducted a retrospective, descriptive study of 24 patients (23 men and 1 woman) whose average age was 49 years and who had undergone bullectomy between 2004 and 2013. Collected data were: factors favoring the occurrence of bullous emphysema, the circumstances in which bubble was detected, data from the radiological examinations, data from pulmonary and cardiovascular function testing, data from bullectomy, data from postoperative clinical and functional examination. Morbidity rate was 37.5 %. The main complication was persistent air leak (7 cases). One patient died two days after surgery due to acute respiratory failure. The average follow-up period was 26 months during which we observed improvement of dyspnea in all patients and no complications. Bullectomy is an effective, reliable and safe surgical technique that may improve patients’ quality of life for a few years.

## Introduction

La bulle d'emphysème est définie comme une distension supérieure à 1cm d'un espace aérien situé au-delà des bronchioles terminales [1]. Elle peut se développer au sein d'un parenchyme pulmonaire sain ou coexister avec un emphysème pulmonaire diffus (panlobulaire ou centrolobulaire), un emphysème péribronchiolaire ou un emphysème paracicatriciel [2, 3]. La chirurgie d'exérèse de ces bulles ou bullectomie est le principal moyen thérapeutique de cette pathologie [4]. Elle est généralement réservée aux patients dont les bulles sont compliquées ou sont à l'origine d'une dyspnée invalidante [4-6]. Le but de notre étude était de déterminer les indications de la bullectomie et d'évaluer les résultats de cette chirurgie dans notre service.

## Méthodes

**Figure 1:**
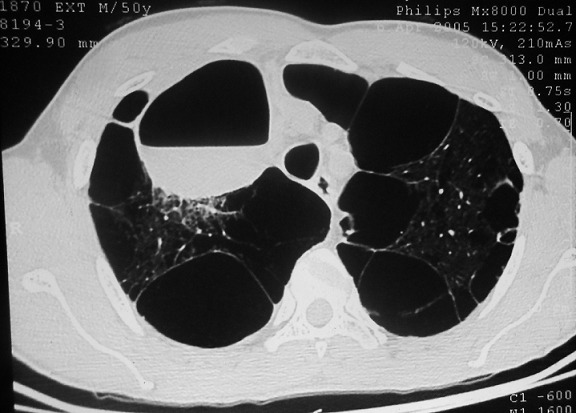
Coupe scannographique montrant une bulle d’emphysème avec un niveau hydro-aérique

**Figure 2:**
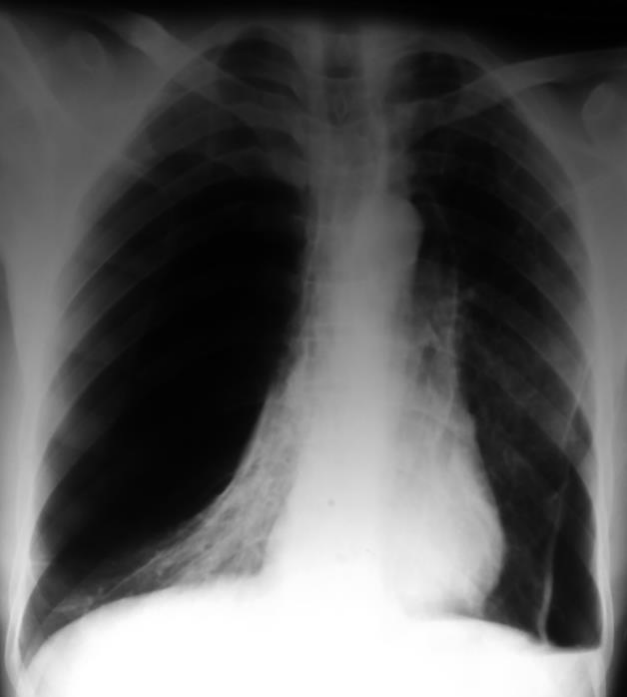
Radiographie du thorax montrant une bulle d’emphysème géante droite

**Figure 3:**
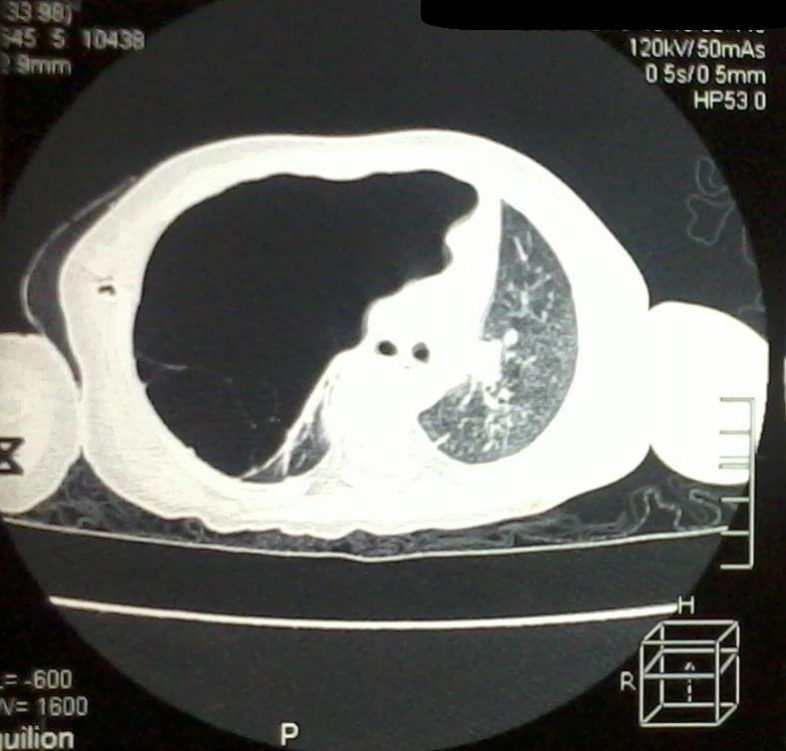
Coupe scannographique montrant une bulle d’emphysème comprimant le poumon sous-jacent et refoulant le médiastin

Il s'agissait d'une étude rétrospective menée dans le service de Chirurgie Thoracique et Cardio-vasculaire du Centre Hospitalier National Universitaire de FANN, à Dakar, sur une période de 10 ans, allant de 2004 à 2013, dans laquelle ont été inclus tous les patients ayant bénéficié d'une bullectomie. Durant cette période d'étude, 24 patients dont 23 étaient de sexe masculin ont bénéficié d'une chirurgie d'exérèse des bulles. Leur moyenne d'âge était de 49 ans (extrêmes de 24 ans et de 81 ans). Une notion de tabagisme actif et un antécédent de tuberculose pulmonaire étaient retrouvés respectivement chez 19 et 5 patients. Les circonstances ayant conduit à la découverte de la bulle d'emphysème sont résumées dans le Tableau 1. La radiographie et le scanner thoracique avaient permis de mettre en évidence les caractéristiques des bulles résumées dans le Tableau 2. Les Figure 1, Figure 2 et Figure 3 montrent respectivement Une coupe scannographique d'une bulle d'emphysème avec un niveau hydro-aérique, une radiographie du thorax d'une bulle d'emphysème géante droite et une coupe scannographique d'une bulle d'emphysème comprimant le poumon sous-jacent et refoulant le médiastin. Une spirométrie avait été effectuée chez 17 patients. Le VEMS moyen préopératoire était de 2 l/seconde avec des extrêmes de 0,75 l/sec et de 3,04 l/sec. Le rapport VEMS/CVF préopératoire était compris entre 0,35 et 0,91 (moyenne de 0,71). L'électrocardiogramme (16 patients) et l'échographie cardiaque (6 patients) n'ont pas retrouvé de signes en faveur d'un cœur pulmonaire chronique. Le drainage préopératoire a été effectué chez 12 patients, pour un pneumothorax. En outre, un patient avait bénéficié, du drainage en urgence d'une bulle compressive associée à une dyspnée d'effort stade 4 de Sadoul. La durée moyenne du drainage préopératoire était de 21 jours, avec des extrêmes de 11 jours et de 35 jours.

**Tableau 1:** Circonstances de découverte de la bulle d’emphysème

Signes fonctionnels	Nombre (%)
Dyspnée d’effort progressive	7 (29,2%)
Stade 2	2 (8,3%)
Stade 3 ou 4	5 (20,8%)
Surinfection de la bulle	1 (4,2%)
Pneumothorax	16 (41,7%)
1^er^ épisode	7 (29,2%)
2^e^ ou 3^e^ épisode	9 (37,5%)

**Tableau 2:** Caractéristiques radiologiques des bulles d’emphysème.

Variable	Nombre (%)
Latéralité	
Droite	6 (25%)
Gauche	5 (20,8%)
Bilatérale	13 (54,2%)
Nombre	
Unique	4 (16,7%)
Multiples	20 (83,3%)
Bulle géante compressive	7 (29,2 %)
Bulle avec un niveau hydro-aérique dans la bulle	1 (4,2%)
Bulle sur emphysème pulmonaire diffus	14 (58,3%)

**Figure 4:**
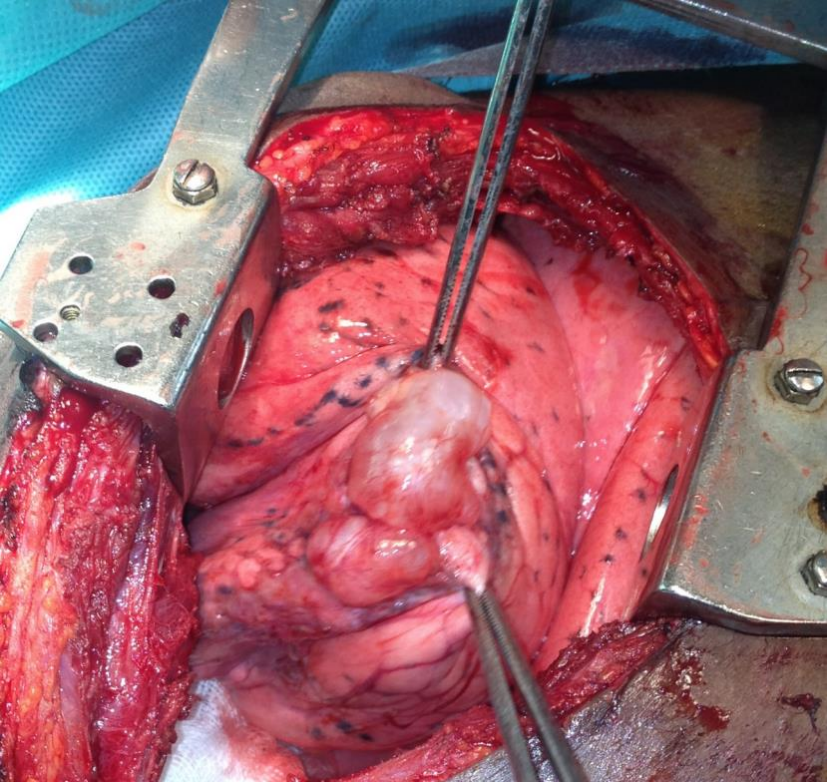
Vue peropératoire de bulles d’emphysème développées au sein d’un parenchyme pulmonaire sain

Les indications de la bullectomie étaient une bulle compliquée d'un pneumothorax (16 patients), une bulle surinfectée (1 patient) et une bulle compressive (7 patients). La voie d'abord était une thoracotomie postéro latérale dans 13 cas, une thoracotomie latérale dans 9 cas et une thoracotomie axillaire dans 2 cas. La Figure 4 est une vue peropératoire de bulles d'emphysème développées au sein d'un parenchyme pulmonaire sain. Chez 21 patients, la bullectomie a consisté en une résection de la bulle sur une pince, suivie d'une suture de la tranche de section par un surjet de Blalock, avec un fil résorbable de calibre 2/0 à aiguille ronde. Dans les autres cas, elle a consisté en une résection par agrafage à l'aide d'une pince mécanique à auto suture. La suture de la tranche de section avait été renforcée par un lambeau de plèvre pariétale chez 8 patients. Un seul patient avait bénéficié d'une bullectomie bilatérale. Celle-ci avait été réalisée un an après la première bullectomie, pour un pneumothorax. La bullectomie a été associée à une pleurectomie chez les patients qui avaient présenté un pneumothorax. Le patient dont le scanner thoracique avait permis d'identifier une masse pulmonaire avait bénéficié d'une biopsie de ladite masse. La durée moyenne du drainage post opératoire était de 10 jours (extrêmes de 7 et 30 jours). La spirométrie de réévaluation a été réalisée dans un délai moyen de 65 mois après la chirurgie (extrêmes de 36 mois et de 114 mois). Elle a été effectuée avec un spiromètre Microlab Spiro^®^355. Le test de Wilcoxon avait été utilisé pour comparer les variations des résultats de la spirométrie après et avant la chirurgie.

## Résultats

La durée moyenne d'hospitalisation était de 22 jours (extrêmes de 9 et 55 jours). Le taux de morbidité était de 37,5 %. La morbidité était représentée par une fuite aérienne persistante (7 cas) un empyème (2 cas), une infection du site opératoire superficielle (2 cas). Les deux patients ayant présenté un empyème post opératoire ont eu des durées de séjour de 34 et de 55 jours. Cet empyème a été traité par une antibiothérapie ciblée associée au drainage thoracique. La fuite aérienne persistante a également été traitée de façon conservatrice, car aucun patient n'a été réopéré pour cette complication. Un patient est décédé au deuxième jour post opératoire. Le décès était dû à une insuffisance respiratoire aiguë. La durée moyenne du suivi post opératoire était de 26 mois [1 mois-9 ans]. Ce suivi a concerné 17 patients (70,8%). Le patient porteur d'une tumeur pulmonaire, dont la biopsie avait conclu à un carcinome épidermoïde moyennement différencié, est décédé trois mois après la chirurgie. Cinq patients ont été perdus de vue moins d'un mois après leur sortie de l'hôpital. Le suivi postopératoire était axé sur la surveillance de la dyspnée et la survenue d'une complication. Nous avons ainsi observé une amélioration de la dyspnée chez tous les patients et nous n'avons noté aucune complication. En effet, dans un premier temps, nous avons observé un amendement de la dyspnée chez tous les patients. Puis dans un second temps, 4 patients ont présenté une dyspnée d'effort. Cette dyspnée était apparue entre la première et la deuxième année suivant la bullectomie. Elle a été classée au stade 1 et au stade 2 de Sadoul respectivement chez trois et un patient. Le VEMS moyen de réévaluation était de 1,81 litres/seconde soit un pourcentage théorique moyen de 67,5%. Le rapport VEMS/CVF de réévaluation était de 0,66. Les résultats de la spirométrie de réévaluation sont comparés avec ceux de la spirométrie préopératoire dans le Tableau 3.

**Tableau 3:** Evolution des paramètres de la spirométrie

	Préopératoire	Réévaluation	Variation	p value	
**VEMS**	2,01	1,81	-0,20	0,41	NS*
**% VEMS prédictif**	61,29	67,50	6,21	0,34	NS*
**CVF**	2,85	2,73	-0,11	0,30	NS*
**% CVF**	70,06	83,25	13,19	0,20	NS*
**VEMS/CVF**	0,71	0,66	-0,04	0,66	NS*

* NS : variation non significative

## Discussion

Les bulles d'emphysème sont plus fréquentes chez les patients de sexe masculin, dont l'âge moyen est généralement supérieur à 40 ans comme dans notre série [7-13]. Le principal facteur étiologique retrouvé par la plupart des auteurs est le tabagisme actif et concerne essentiellement les sujets de sexe masculin [9, 12, 13]. Dans les pays d'endémie tuberculeuse comme le nôtre, la tuberculose constitue le second facteur étiologique [12, 13]. La dyspnée d'effort progressive constitue pour certains auteurs la principale manifestation clinique des bulles d'emphysème [7, 12, 13]. Tandis que pour d'autres auteurs, comme dans notre série, c'est le pneumothorax qui constitue la principale manifestation clinique [10, 14]. Les autres manifestations, moins fréquentes sont la surinfection de la bulle et l'hémoptysie [6, 8, 15]. Le VEMS et le rapport VEMS/CVF moyens préopératoires dans notre série sont supérieurs à ceux des séries de Krishnamohan *et al.*, Gunnarsson *et al.*, Lone *et al.*, De Giacomo *et al.* Dans ces séries, le nombre de patients ayant une BPCO est par contre plus élevé que dans la nôtre [8-10, 16]. Le drainage préopératoire dans notre série avait été effectué pour lever la compression du parenchyme sain par la bulle géante ou par le pneumothorax, comme le préconise Le Pimpec-Barthes *et al.* [5] Tous les patients de notre série, comme dans la plupart des séries récentes sont symptomatiques. En effet, il est actuellement conseillé de réaliser une bullectomie chez les patients symptomatiques, chez les patients dont la bulle d'emphysème est compliquée ou chez les patients dont la bulle occupe plus du tiers d'un hémithorax [4, 5, 17]. Les patients asymptomatiques doivent faire l'objet d'une surveillance clinique et radiologique. Lors de cette surveillance, si le volume de la bulle augmente, l'indication d'une résection pourra être prise [4]. La vidéothoracoscopie est actuellement la voie d'abord préférentielle [4, 5]. Elle a l'avantage d'être peu invasive et s'est avérée aussi fiable et sûre que la thoracotomie [4, 5, 16, 18]. La bullectomie par agrafage à l'aide d'une pince à autosuture est la technique de bullectomie la plus courante [16, 18]. Les autres techniques sont la ligature de bulles à l'aide de boucles préformées *(Endoloop^®^),* la coagulation de la bulle au bistouri électrique ou au laser, ou l'injection de colle biologique [5, 19].

Lorsque la résection de la bulle par agrafage est faite sur un poumon emphysémateux, la ligne de suture peut être renforcée par un matériel prothétique tel que du péricarde bovin ou du polytétrafluoroethylène ou PTFE [8, 20]. Dans notre pratique quotidienne, l'inaccessibilité financière de ces moyens et le niveau de maîtrise de cette technique constituent les principaux obstacles à l'utilisation de la voie endoscopique. Aussi, tous nos patients sont opérés par thoracotomie et la résection sur pince de la bulle d'emphysème suivie de la suture de la tranche de section par un surjet de Blalock remplace l'agrafage automatique. Le renforcement de la suture de la tranche de section par un lambeau pleural constitue l'alternative au péricarde bovin ou au PTFE. En dehors du renforcement de la suture de la tranche de section, le second moyen utilisé pour réduire les fuites aériennes persistantes était la pleurectomie. Il est également réalisé par d'autres auteurs [8, 18]. Il permettrait la réduction des fuites aériennes persistantes et la prévention de la survenue ultérieure d'un pneumothorax en favorisant une symphyse pleurale. La principale complication après la bullectomie est la fuite aérienne persistante. Dans notre série, sa fréquence est de 29%. Dans la littérature, selon que le parenchyme adjacent à la résection de la bulle est emphysémateux ou non, sa fréquence varie de 5 à 75 % [7, 8, 10, 16, 18]. La présence d'une fuite aérienne contribue à allonger la durée du drainage postopératoire et la durée d'hospitalisation. Ainsi, les durées maximales d'hospitalisation dans les séries de shah *et al.*, Gunnarson *et al.*, Krishnamohan *et al.*, sont respectivement de 41 jours, 57 jours et de 74 jours [8, 9, 11]. L'infection de la plaie opératoire et l'empyème sont des complications retrouvées dans d'autres séries [10, 16]. Le taux de mortalité de notre série est supérieur à celui de plusieurs auteurs [8-10, 16, 18]. Des auteurs tels que Menconi *et al*. ou Greenberg *et al.* rapportent des taux plus élevés qu'ils attribuent à la présence d'un emphysème pulmonaire diffus [17, 21]. De nombreuses études ont montré qu'après la bullectomie, on observe généralement une régression de la dyspnée et une amélioration du VEMS et du rapport VEMS/CVF durant les trois premières années [8, 9, 16, 18]. Gunnarsson *et al.* , comme nous, rapporte une détérioration des paramètres spirométriques qui reviennent aux valeurs de base préopératoires dès la cinquième année [8].

## Conclusion

La bullectomie est une technique chirurgicale efficace, fiable et sûre qui peut permettre aux patients d'avoir une meilleure qualité de vie pendant quelques années. Il convient toutefois de bien sélectionner les patients devant bénéficier d'une bullectomie afin de réduire les taux de morbidité et de mortalité de cette chirurgie.

### Etat des connaissances actuelles sur le sujet

- La bullectomie est indiquée chez les patients porteurs d'une bulle d'emphysème symptomatique;
- La voie d'abord chirurgicale préconisée doit être la moins invasive possible, à savoir la vidéothoracoscopie;
- Les fuites aériennes persistantes constituent l'une des complications majeures de cette chirurgie.

### Contribution de notre étude à la connaissance

- L'emphysème bulleux est une entité nosologique réelle en Afrique en général et en Afrique subsaharienne en particulier;
- La bullectomie est réalisable dans notre contexte, c'est-à-dire par thoracotomie, avec des résultats satisfaisants.

## Conflits d’intérêts

Les auteurs ne déclarent aucun conflit d'intérêt.
